# Paragonimiasis in Children in Southwest China: A retrospective case reports review from 2005 to 2016

**DOI:** 10.1097/MD.0000000000007265

**Published:** 2017-06-23

**Authors:** Zongrong Gong, Ruixue Miao, Min Shu, Yu Zhu, Yang Wen, Qin Guo, Qiong Liao, Chaomin Wan

**Affiliations:** Department of Pediatric Infectious Disease, West China Second University Hospital/West China Women's and Children's Hospital, Sichuan University, Chengdu, Key Laboratory of Birth Defects and Related Diseases of Women and Children, Ministry of Education (Sichuan University) China.

**Keywords:** children, china, diagnosis, paragonimiasis

## Abstract

**Background:**

Paragonimiasis infection has no specific symptoms or typical radiologic findings, leading to the possibility of misdiagnosis. Thus, the objective of this study was to analyze clinical and radiological features, and treatment outcome of paragonimiasis in children in Southwest China to improve the awareness of this disease.

**Methods:**

We retrospectively reviewed the records of children diagnosed with paragonimiasis in West China Second University Hospital between 2005 and 2016. The confirmed diagnosis of paragonimiasis was based on epidemiology history and seropositivity for paragonimiasis and/or detection of paragonimus eggs. Clinical, laboratory, and imaging findings of patients were examined in order to summarize risk factors, clinical characteristics, and treatment outcomes of these patients.

**Results:**

A total of 123 patients were included; of them 112 (91.1%) lived in villages and 72 (58.5%) had a history of consuming freshwater crabs. Patients with paragonimiasis most frequently showed respiratory symptoms, including cough (26.0%, 32/123) and tachypnea (16.3%, 20/123), and gastrointestinal symptoms, including abdominal pain (26.8%, 33/123), abdominal distention (22.8%, 28/123), and vomiting (13.0%, 16/123). Laboratory examination showed elevated white blood cell (WBC) counts in the peripheral blood in 89 (72.4%) patients and eosinophilia in 102 (82.9%) patients. Tuberculosis (TB) coinfection was found in 4 (3.3%) patients. Main imaging findings included: effusions (90.4%), lymphadenopathy (40.4%), pulmonary ground-glass opacities (36.2%), cystic lesions (18.1%), and pleural thickening (17.0%). Twenty-nine patients (23.6%) received more than 1 course of praziquantel (PZQ). Additionally, 4 (19.0%) of 21 patients who were discharged from the hospital without complete treatment required rehospitalization for residual serous effusions. Moreover, patients from pericardial effusion group showed longer hospital stays and less elevated WBC counts than those from nonpericardial effusion group.

**Conclusion:**

Paragonimiasis should be considered in patients from endemic areas, especially in those with gastrointestinal and/or respiratory symptoms, elevated WBC count, eosinophilia, and serous effusions. Additionally, longer hospital stay may be necessary in cases of paragonimiasis associated with pericardial effusions.

## 1 Introduction

Paragonimiasis, also known as lung fluke disease, is a parasitic disease found in humans and other mammals that is caused by a trematode of the genus *Paragonimus* via ingestion of raw, inadequately cooked crabs or crayfish infected with *Paragonimus metacercariae.*^[1]^ The parasite is endemic in Southeast Asia, the Indian subcontinent, South and North America, and Africa.^[2,3]^ It is estimated that about 293.8 million people are at risk of infection with *Paragonimus* parasites, 195 million of them residing in China.^[4,5]^ More than 50 species have been described all over the world, among which the most predominant in China are *P westermani* and *P skrjabini*.^[3,5,6]^ Meanwhile, paragonimiasis is prevalent in most of the Sichuan basin, especially in the eastern part of Sichuan province and Chongqing city.^[5,7]^

Previously, paragonimiasis was endemic to villages.^[8]^ Recently, due to transportation and economic development, more patients with paragonimiasis come from cities^[9]^; therefore, special attention is necessary in this category of patients. Moreover, the diagnosis of paragonimiasis is difficult due to nonspecific clinical symptoms, particularly in children who cannot accurately describe their symptoms and dietary history. Additionally, it has been recently reported that paragonimiasis associated with effusions have unsatisfactory response to the initial praziquantel (PZQ) treatment in adults.^[10,11]^ Paragonimiasis has been rarely reported in children and usually was misdiagnosed as tuberculosis (TB), pneumonia, liver abscess, or carcinoma.^[12–14]^ Better diagnostic methods should be developed for paragonimiasis to enable rapid treatment. Thus, to improve the awareness of this disease, we investigated the clinical and imaging features, and treatment outcome of paragonimiasis in children in Southwest China. We retrospectively analyzed the records of patients diagnosed with paragonimiasis in our hospital between 2005 and 2016. We summarized the clinical characteristics and treatment outcomes of these patients, as well as analyzed the risk factors of patients with pericardial effusion by comparing the difference between patients with pericardial effusion (pericardial effusion group) and those without pericardial effusion (nonpericardial effusion group).

## 2 Methods

Southwest China is an endemic area for paragonimiasis.^[5,7]^ We analyzed the records of patients diagnosed with paragonimiasis between May 2005 and May 2016 in the Department of Pediatric Infectious Disease, West China Second Hospital, Sichuan University (Chengdu, China). This tertiary-care, university-affiliated hospital for women and children serves 20,000 children inpatients from Southwest China annually, providing treatment for severe diseases, in addition to referral, consultation, and other large clinical work for women and children from Sichuan Province, as well as Southwest China. Paragonimiasis was suspected in any patient who has been living in, or has traveled to an endemic region, who has consumed raw freshwater crab or crayfish and who has developed clinical symptoms (such as abdominal pain, vomiting, cough, and dyspnea) consistent with paragonimiasis. Subsequently, aparasite-specific serologic test for paragonimiasis by using micro-enzyme linked immunosorbent assay (ELISA)^[15]^ was performed in suspected patients by the Center for Disease Control and Prevention, Sichuan province. The confirmed diagnosis of paragonimiasis was based on epidemiology history and seropositivity for paragonimiasis and/or detection of paragonimus eggs (in sputum, feces, effusion, or biopsy). The time to diagnosis (considered from the moment when the patient first noticed symptoms to the date of diagnosis of paragonimiasis) was recorded.

The white blood count (WBC) and eosinophil counts were obtained by standard procedures. A systematic review of chest radiographs or computed tomography (CT), abdomen CT or ultrasonography, and magnetic resonance imaging or CT of the brain was performed in order to detect any effusion, lymphadenopathy, pulmonary ground-glass opacities, cystic lesions, pleural thickening, hepatomegaly, pulmonary nodular opacities, or other abnormalities.

Thoracentesis, abdominocentesis, or pericardiocentesis was performed in patients with effusions to exclude other diseases. Additionally, bone marrow aspiration, cerebrospinal fluid analysis, and biopsy were performed when necessary to rule out other diseases, such as hematological malignancies, infections, or cancers. The smears obtained from sputum, stool, or effusion of patients were stained for cytology; additionally, they were examined for the presence of eggs, stained with Ziehl–Neelsen stain, and cultured for acid-fast bacilli.

All patients were treated with PZQ at a dose of 75 mg/kg/day for 3 days. Hematological and serological examinations were performed after treatment to evaluate patient recovery from the infection. Another course of PZQ was used in patients with unsatisfactory responses, persistent symptoms, or pulmonary involvement. We analyzed the risk factors of pericardial effusion by comparing the differences between pericardial effusion and nonpericardial effusion groups.

SPSS statistical software version 20.0 (IBM corp., Armonk, NY) was used to compare the differences of characteristics between the pericardial effusion and nonpericardial effusion groups. Measurements were presented as means ± standard deviation and were analyzed by using independent sample *t* test for continuous variables and χ^2^ test or Fisher exact test for categorical variables. *P*-values less than .05 were considered statistically significant.

Ethical approval for this study was approved by the medical committee of West China Secondary Hospital, due to the retrospective nature of the study, informed consent was waived.

## 3 Results

### 3.1 Clinical manifestations

**Figure 1:**
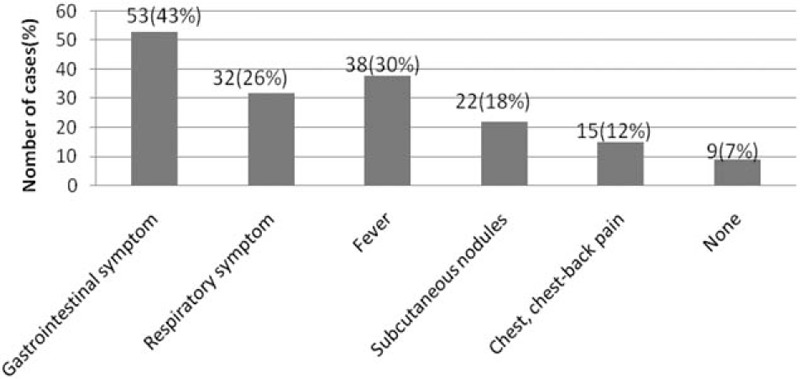
Symptoms of paragonimiasis. Gastrointestinal symptoms included abdominal pain, abdominal distention, nausea, and vomiting. Respiratory symptoms included sputum and tachypnea. Nine (7%) patients with no symptoms were diagnosed with paragonimiasis based on incidentally detected eosinophilia during the check-up for other diseases. Note that the total number of patients is more than 123 because some of them presented more than one symptom.

Over a period of 11 years, 123 patients (87 male and 36 females) with a median age of 7 years (range, 1–13 years) were diagnosed with paragonimiasis. Of the patients, 91.1% (112/123) lived in villages and 8.9% (11/123) lived in cities. The median time to diagnosis was 30 days (range, 2 days–5 years). The prevalence of a history of freshwater crab consumption was 58.5% (72/123), nonavailable dietary history was 30.9% (38/123), and the remaining 10.6% (13/123) of patients or their guardians denied the consumption of freshwater crabs. The most common reasons for seeking medical care were gastrointestinal symptoms, including abdominal pain (26.8%, 33/123), abdominal distention (22.8%, 28/123), vomiting (13.0%, 16/123), and respiratory symptoms, including cough (26.0%, 32/123) and tachypnea (16.3%, 20/123). The symptoms of patients are summarized in Figure 1.

### 3.2 Laboratory findings

Elevated peripheral blood WBC (>10^3^/mm^3^) was present in 89 (72.4%) patients, and peripheral blood eosinophilia (>7.0%) was detected in 102 (82.9%) patients at admission. Four (3.3%) patients showed positive results for acid-fast bacilli infection. *Paragonimus* was detected by the presence of eggs in 1 (0.8%) patient. We found that the length of hospital stay was longer, and WBC was less elevated in the pericardial effusion group. The characteristics of the pericardial effusion and nonpericardial effusion groups are summarized in Table 1.

**Table 1:**
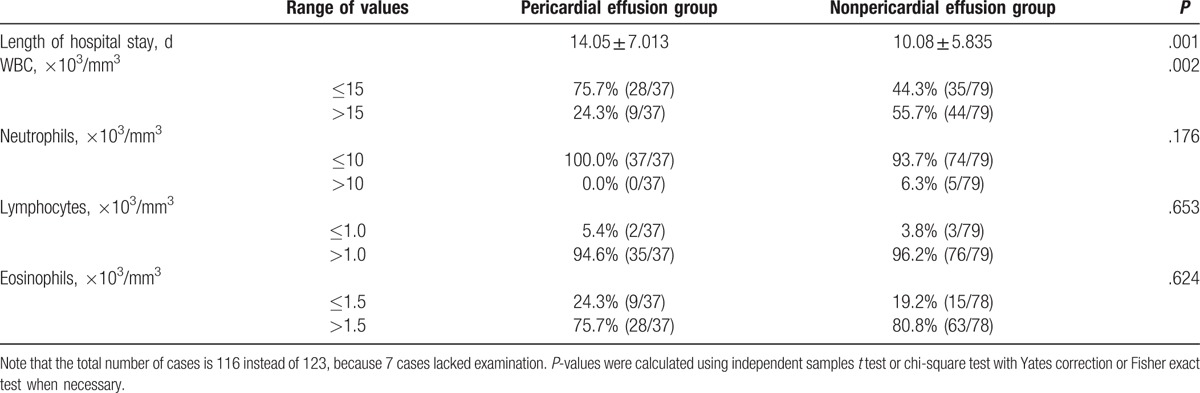
The characteristics of the pericardial effusion groups and nonpericardial effusion groups.

### 3.3 Imaging findings

We retrospectively reviewed the imaging findings of 94 patients. In the remaining 29 cases, imaging examination was not performed. Effusion (in pleura, peritoneum, pericardium, or pelvis) was found in 85 (90.4%) patients, lymphadenopathy in 38 (40.4%) patients, and pulmonary ground-glass opacities in 34 (36.2%) patients. Detailed imaging findings are presented in Table 2.

**Table 2:**
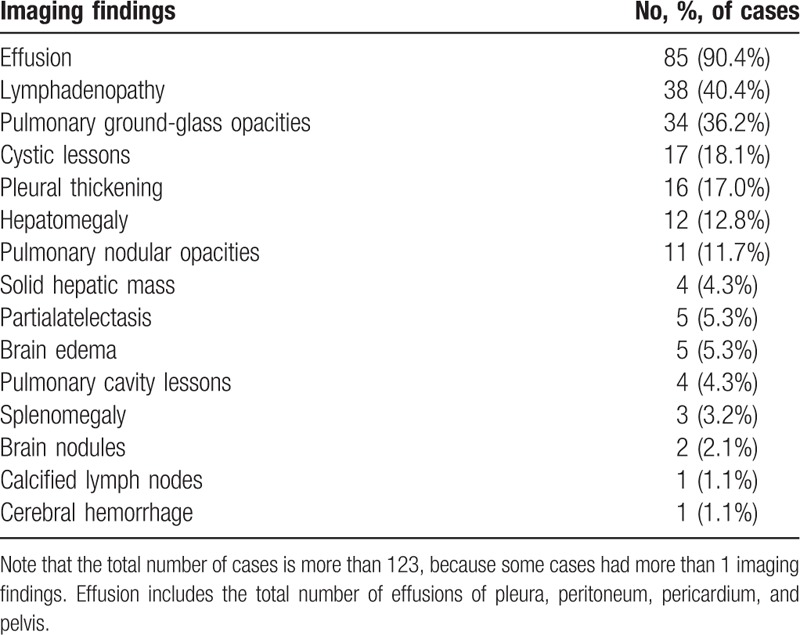
The characteristics of imaging findings.

### 3.4 Examination of effusion

The samples of pleural (n  =  24), peritoneal (n  =  4), pericardial (n  =  25), and cerebrospinal (n  =  11) fluid were obtained by punctures. Among them, paragonimus ova were found in 1 patient. Bone marrow aspiration was performed in 20 patients (16.3%); the samples of 18 (90.0%) patients showed high eosinophil counts, while the remaining 2 patients had normal bone marrow aspiration results. Most samples of serous fluid appeared grossly turbid and yellowish (n  =  40/52, 76.9%), while in the rest of patients, serous fluid was red or faint red. Most samples showed a high positivity rate of the Rivalta test (94.3%), high red blood cells counts (92.5%), eosinophilia (98.0%), high total protein levels (96.1%), high lactate dehydrogenase levels (88.2%), and low glucose levels (68.9%). The main results of clinical and laboratory examinations of effusion are summarized in Table 3.

**Table 3:**
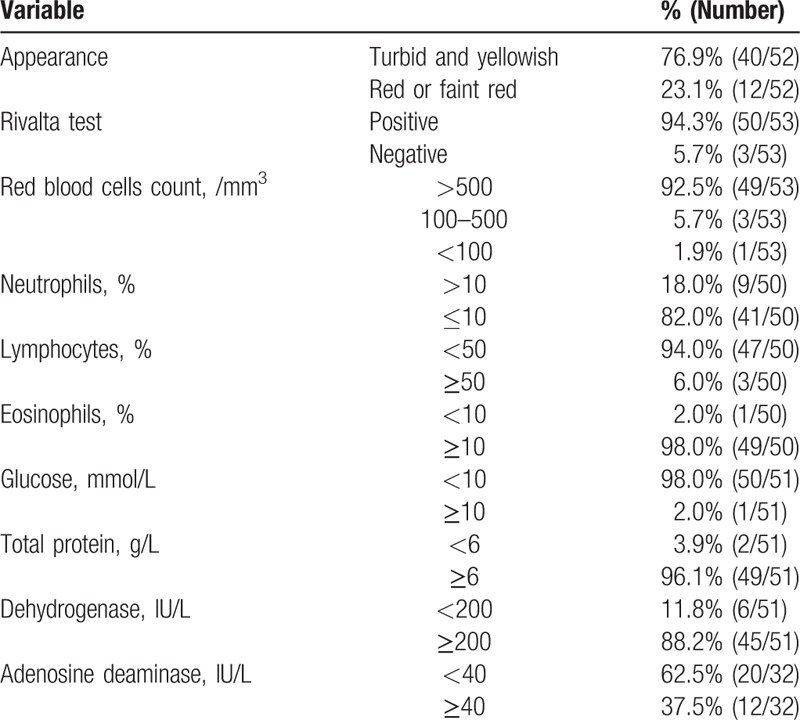
Results of clinical and laboratory examination of effusion.

### 3.5 Treatment and outcomes

All patients were initially treated with PZQ for 3 days. Ninety-four patients (76.4%) showed improvement in symptoms. However, 29 (23.6%) patients required another course of PZQ treatment. Twenty-one (17.1%) patients could not adhere to the treatment and were discharged without complete treatment; among them, 4 (19.0%) patients required rehospitalization for residual effusions. The average length of hospital stay was 11.23 ± 6.63 days.

## 4 Discussion

Paragonimiasis, like all trematode infections, has a parasitic cycle, requiring 2 intermediate hosts: first is rever snail, where the embryonated eggs become carcariae, and then a crayfish or freshwater crab, where they evolve to matacercariae, which are passed to the definitive host (human being or carnivorous mammal) when these crustaceans are ingested in an uncooked state.^[16]^ Immature forms migrate through the duodenal wall, peritoneal cavity, and diaphragm to become encapsulated and mature within the pulmonary parenchyma,^[1]^ ectopic infections can occur in unexpected regions, including the liver, brain, muscle, and subcutaneous tissues.^[17–19]^ Therefore, symptoms of paragonimiasis were usually variable, as agents of a “neglected tropical disease.”^[20]^ This study is one of the largest retrospective studies investigating the paragonimiasis in children. This study concluded the clinical and imaging findings of paragonimiasis and its treatment course to improve the awareness of this disease.

In this study, 30.9% of children had no history of freshwater crab or crayfish consumption, while 10.6% of children denied freshwater crab consumption. Our findings are consistent with those of a Japanese 12-year retrospective paragonimiasis case review^[21]^ that found no available history of freshwater crab consumption in 29.5% (128/434) of adult patients and no history in 30.6% (11/36) of patients, and with those of a Korean PW study^[22]^ that found no available history in 22.2% (8/36) of patients. Therefore, paragonimiasis cannot be excluded when there is no history of freshwater crab consumption or no available history, especially in children who cannot exactly describe and may forgot dietary history. A previous report^[23]^ indicated that children catching and eating raw crustaceans while playing or helping their parents in agricultural activities are at highest risk of infection. In this study, 8.9% of patients lived in cities. This fact might be associated with economic development and with the sale of live freshwater crabs in supermarkets in cities. One study showed that 39.6% of freshwater crabs in Three Gorge Reservoir Region were infected with *P metacercariae.*^[7]^ This result implies that an increased awareness of paragonimiasis is necessary in children with clinical symptoms, even in those from cities or denying freshwater crab consumption.

We found that the symptoms of paragonimiasis were usually variable and nonspecific, including fever, cough, abdominal pain, abdominal distention, nausea, and vomiting. Similar results were reported by previous studies.^[13,21]^ Respiratory symptoms of can be easily confused with those of TB.^[12,24]^ Additionally, Barennes et al^[25]^ reported that 3 (2.5%) of 118 patients with negative acid-fast bacilli smear who were suspected to have pulmonary TB were diagnosed with paragonimiasis. However, no report about paragonimiasis patients coinfected with TB is available. In our study, 4 (3.3%) of 123 patients had positive acid-fast bacilli results. Therefore, special attention should be paid to the fact that paragonimiasis patients that come from TB endemic areas could be coinfected with TB.

The definitive diagnosis of paragonimiasis is based on the presence of eggs in patients’ feces or sputum, or flukes in histological specimens. We detected paragonimus eggs in 0.8% (1/123) of patients. Our findings differ from those reported by Jeon, Kyeongman et al^[22]^ who detected paragonimus eggs in 46% (12/26) of Korean adult patients. This difference could be explained by the fact that the median time to diagnosis was 30 days in our study. However, these eggs are not present until 2 to 3 months after infection.^[5]^ Therefore, a more sensitive and specific diagnosis method is necessary. ELISA is the ideal method for the diagnosis of paragonimiasis, as it has 90.2% sensitivity and 100.0% specificity.^[15]^ Meanwhile, it has been used to confirm the diagnosis paragonimiasis.^[5,7,22]^ Hence, paragonimiasis was diagnosed in this study based on ELISA paragonimus-specific serologic test and/or paragonimus eggs detection (in sputum, feces, effusion, or biopsy).

Eosinophilia in peripheral blood, serous fluid, and bone marrow was commonly observed in our patients. These results agree with those reported by Hwang et al^[12]^ who found eosinophilia in pleural fluid in 82.4%(14/17) of patients. Moreover, 73 (59.3%), 38 (30.9%), 38 (30.9%), and 9 (7.3%) patients had pleural, peritoneum, pericardium, and pelvic effusions, respectively. These results may be associated with the immunologic sensitization and immunologic response to parasites. This may be an indication to suspect paragonimiasis in patients with nonspecific symptoms.

PZQ and albendazole are 2 drugs recommended by World Health Organization as mainstay treatment for human pulmonary paragonimiasis. However, a recent report showed that patients with effusion and had longer duration of respiratory symptoms, higher ELISA titers for pulmonary paragonimiasis, and/or multiple pulmonary lesions needed additional PZQ treatment.^[10]^ Obara et al^[11]^ reported that 16.7% (3/18) of patients needed additional PZQ treatment. We found that 29 (23.6%) patients needed more than 1 course of PZQ. These results are similar to those reported by Oh et al^[10]^ who showed that 25% of patients with PW needed additional PZQ treatment. Additionally, 4 (19.0%) of 21 patients who were discharged without complete treatment required rehospitalization, which suggests that all patients should be cured in accordance with current clinical standards.

On the analysis of patients with pericardial effusions, we observed longer hospital stay and less elevated WBC in the pericardial effusion group, which was not reported in previous studies. This may indicate that patients with pericardial effusion may need longer hospital treatment, although their probability of severe infection is lower. Therefore, the awareness of pericardial effusion treatment should be increased among clinicians.

The present study had certain limitations. Due to the 11-year period, it was difficult to know whether ELISA-detected antibodies were acute (IgM) or convalescent (IgG). Additionally, patients came from a single facility located in Southwest China; therefore, the findings of this study could not represent the whole China. Future studies should investigate paragonimiasis in the whole China, including the South and North.

In conclusion, when children from endemic areas (cities or villages) present with gastrointestinal or respiratory symptoms, and physical or imaging examinations show serous effusion, an ELISA test should be performed to provide rapid diagnosis and treatment of paragonimiasis in order to avoid severe complications. One standard course of PZQ is effective in 76.4% of patients, while the remaining patients may need an additional course of PZQ. Additionally, our study found that pericardial effusion can increase the duration of hospital stay, although the possibility of severe infection is less in such patients. Therefore, physicians should be aware of this disease and its early diagnosis and treatment.

## Acknowledgments

The authors thank the guardians of patients for permitting to use patient's data. The authors also thank Editage (www.editage.com) for English language editing.
